# Prevalence of scarred and dysfunctional myocardium in patients with heart failure of ischaemic origin: A cardiovascular magnetic resonance study

**DOI:** 10.1186/1532-429X-13-53

**Published:** 2011-09-21

**Authors:** Christos V Bourantas, Nikolay P Nikitin, Huan P Loh, Elena I Lukaschuk, Nassar Sherwi, Ramesh de Silva, Ann C Tweddel, Mohamed F Alamgir, Kenneth Wong, Sanjay Gupta, Andrew L Clark, John GF Cleland

**Affiliations:** 1 Department of Cardiology, Academic Unit, University of Hull, Postgraduate Medical Institute, Kingston-upon-Hull, UK

**Keywords:** Heart failure, Myocardial infarction, Hibernation, Cardiovascular magnetic resonance imaging, Late gadolinium enhancement

## Abstract

**Background:**

Cardiovascular magnetic resonance (CMR) with late gadolinium enhancement (LGE) can provide unique data on the transmural extent of scar/viability. We assessed the prevalence of dysfunctional myocardium, including partial thickness scar, which could contribute to left ventricular contractile dysfunction in patients with heart failure and ischaemic heart disease who denied angina symptoms.

**Methods:**

We invited patients with ischaemic heart disease and a left ventricular ejection fraction < 50% by echocardiography to have LGE CMR. Myocardial contractility and transmural extent of scar were assessed using a 17-segment model.

**Results:**

The median age of the 193 patients enrolled was 70 (interquartile range: 63-76) years and 167 (87%) were men. Of 3281 myocardial segments assessed, 1759 (54%) were dysfunctional, of which 581 (33%) showed no scar, 623 (35%) had scar affecting ≤50% of wall thickness and 555 (32%) had scar affecting > 50% of wall thickness. Of 1522 segments with normal contractile function, only 98 (6%) had evidence of scar on CMR. Overall, 182 (94%) patients had ≥1 and 107 (55%) patients had ≥5 segments with contractile dysfunction that had no scar or ≤50% transmural scar suggesting viability.

**Conclusions:**

In this cohort of patients with left ventricular systolic dysfunction and ischaemic heart disease, about half of all segments had contractile dysfunction but only one third of these had > 50% of the wall thickness affected by scar, suggesting that most dysfunctional segments could improve in response to an appropriate intervention.

## Background

Ischaemic heart disease (IHD) is a common cause of left ventricular (LV) systolic dysfunction leading to chronic heart failure (CHF) [1]. Patients with lower LV ejection fraction (EF) and more extensive coronary artery disease have a worse prognosis [2]. LV systolic dysfunction in patients with IHD may be due to either myocardial necrosis leading to scar, or to impaired myocardial contractility despite myocardial viability (hibernation or stunning) [3]. Viable but dysfunctional myocardium can potentially recover if the ratio of myocardial oxygen supply to demand can be improved either by coronary revascularisation or with anti-ischaemic treatment, although recovery of function may take months or even years [4-7]. Many hearts are likely to have systolic dysfunction related to a complex substrate including variable quantities of myocardium affected by full or partial thickness scar, stunning, hibernation, and reversible ischemia.

The proportion of patients who have a substantial volume of myocardium that is dysfunctional but viable is uncertain [8]. The inconsistencies in available data may be related to limitations of the imaging methods conventionally used to detect myocardial viability (myocardial perfusion scintigraphy, positron emission tomography and stress echocardiography) or the populations studied. Cardiovascular magnetic resonance (CMR) with late gadolinium enhancement (LGE) is a high-resolution imaging method that can estimate scar volume and transmurality and provide information regarding myocardial viability. The method is based on the accumulation of paramagnetic contrast (gadolinium) in necrotic (acute infarction) or scar tissue. Myocardial injury without necrosis or scarring does not lead to LGE despite the presence of myocardial hibernation/stunning [9-11]. Given the high spatial resolution of CMR, it is possible to measure not only the number of myocardial segments affected but also the transmural extent of scar, a capability unmatched by other imaging techniques. Scars affecting ≤50% of the thickness of the myocardial wall appear to predict functional improvement following revascularisation or medical therapy [11,12].

Revascularisation might lead to improvement in LV systolic function and improve the clinical state of patients with CHF. However, the potential for revascularisation is dependent upon the extent of reversible ischaemia as opposed to scar in the areas to be revascularised. In addition, the extent and distribution of transmural scar may also affect the response to cardiac resynchronisation therapy (CRT) [13,14]. We therefore designed the present study to investigate the prevalence and distribution of scarred myocardium (and assess the relationship between contractile dysfunction and the extent of myocardial scar) in an epidemiologically-representative group of patients with CHF and IHD, who did not complain of symptoms of angina and in whom revascularisation as a treatment option was not excluded by severe co-morbidities or frailty.

## Methods

### Study subjects

We prospectively enrolled patients with stable clinical signs and symptoms of CHF (New York Heart Association (NYHA) functional class I to III) due to LV ventricular systolic dysfunction and IHD attending a community-based heart failure clinic serving a population of just over 0.5 million. The diagnosis of heart failure was based on symptoms and signs assessed by a cardiologist. Patients with a baseline echocardiogram showing a LVEF < 50% were considered to have systolic dysfunction. The diagnosis of IHD was confirmed by a history of myocardial infarction, coronary revascularisation or > 50% of luminal diameter coronary stenoses on angiography [15].

Exclusion criteria were significant primary valvular or congenital heart disease, a myocardial infarction or revascularisation during the 12 months prior to recruitment, and conventional contraindications to CMR (metal in the eye or in the brain, pacemakers or defibrillators, and claustrophobia). Patients who reported anginal symptoms were also excluded from the study since these patients would be expected to have substantial amounts of viable myocardium subtended by diseased coronary arteries. Patients with severe co-morbidities or who were considered too frail for revascularisation were also excluded.

Patients were first treated with appropriate pharmacological treatment including diuretics, renin-angiotensin-aldosterone system (RAAS) inhibitors, β-blockers and aldosterone antagonists unless contra-indicated or not tolerated and were seen regularly in a heart failure clinic to ensure continued optimisation of treatment and were then referred for CMR. Typically there was a delay (mean ± standard deviation: 4 ± 5.5 months) between initial assessment and CMR during which ventricular function may have improved with therapy. Written informed consent was obtained in all study patients. The study complies with the Declaration of Helsinki and was approved by the local research ethics committee.

### Cardiac magnetic resonance imaging

Patients underwent CMR on a 1.5 Tesla scanner (Signa CV/i, GE Medical Systems) using ECG-triggered breath-hold gradient-echo in steady-state acquisition (FIESTA) imaging. After initial localizing scans, cine LV horizontal long-axis, vertical long-axis and contiguous short-axis images covering the LV from apex to base (slice thickness 10 mm) were obtained. The multi-slice short-axis cine data sets were analyzed by an expert observer whose reliability and reproducibility has already been tested (Table 1) [16]. This observer manually traced the endocardial and epicardial borders in the end-diastolic and end-systolic frames, in each one of the contiguous short-axis slices using specialized software (MEDIS, Leiden, NL). These borders were then used to calculate LV end-diastolic volume (EDV), end-systolic volume (ESV), EF and myocardial mass (MM). The indices of EDV, ESV and MM were obtained by correcting for body surface area. Wall thickening was assessed blind to the LGE results by an expert observer using a 17-segment model as recommended by the American Heart Association [17]. The segments were classified as normal or dysfunctional (mildly hypokinetic, severely hypokinetic, akinetic or dyskinetic) on the basis of visual assessment.

**Table 1:** Intra- and interobserver variability for the left ventricular indices.

	CMR measurements	Intra-observer variability (n = 30)	Inter-observer variability (n = 30)
LVEF (%)	35.6 ± 9.1	0.1 ± 2.7	0.0 ± 2.1
LVEDV index (ml/m^2^)	121 ± 41	1.2 ± 3.7	-1.2 ± 1.3
LVESV index (ml/m^2^)	83 ± 38	0.6 ± 2.8	-1.0 ± 3.2
LVMM index (g/m^2^)	83 ± 22	1.4 ± 2.9	-0.3 ± 3.3

Results are presented as mean ± standard deviation

LV, left ventricle; EF, ejection fraction; EDV, end-diastolic volume; ESV, end-systolic volume index; MM, myocardial mass.

The reproducibility and reliability of the observer who visually evaluated the presence of wall thickening abnormalities was examined in 30 randomly selected CMR scans. The observer reviewed the data twice within 2 months blind to the results of the first scan. A second observer reviewed the same scans once. Intra- and interobserver agreement was high with Cohen's *k *being close to unity (*k = *0.90, *p >*0.001 and *k = *0.88, *p >*0.001, respectively).

### CMR with LGE

A commercially available gadolinium-based contrast agent, gadodiamide (Omniscan, GE Healthcare, Amersham, UK), was injected intravenously at a dose of 0.1 mmol/kg of body weight, and 10-15 min after the injection CMR was performed using a segmented inversion-recovery fast gradient echo sequence that has been described in detail previously [18]. LGE images were acquired in multiple short-axis views identical to those obtained for cine CMR. The planimetric analysis of LGE images was performed automatically using the MASS-PLUS module of MRI-MASS software (MEDIS, Leiden, NL). The software provides a semi-automatic threshold tool that allows the identification of pixels showing signal intensity higher than a pre-defined threshold (> 2 standard deviations above normal myocardium). The same 17-segment model was used with the area of LGE enclosed by the epicardial and endocardial contours measured in 6 basal, 6 mid-cavity and 4 apical segments and the extent of LGE was defined as a percentage of LGE area relative to total segment area [17]. Segments were then graded semi-quantitatively using the following scale: no LGE, 1% to 25%, 26% to 50%, 51% to 75%, and 76% to 100% of wall thickness. Visual estimation of LGE extent in LV horizontal and vertical long-axis views was used for the assessment of the 17^th ^segment (apex).

Potentially viable but dysfunctional myocardial segments were defined as those with impaired thickening but with ≤50% LGE [11]. A patient was classified as having "substantial" myocardial viability if 5 or more such segments were present.

### Statistical analysis

Results are presented as median and interquartile range (IQR) for continuous variables or as numbers (percentages) for categorical variables. Comparisons between sub-groups were made using the Mann-Whitney U test for continuous variables and the chi-square test for categorical variables. Linear regression analysis and the Pearson correlation coefficient was apply to examine the association between LVEF and the number of dysfunctional segments. A *P *value < 0.05 was considered significant.

## Results

### Study subjects

Clinical characteristics of the study population are shown in Table 2. Of 193 patients, only 129 (67%) had suffered a clinically documented myocardial infarction and 71 (37%) had undergone coronary revascularisation prior to initial assessment. No patients had undergone revascularisation between initial assessment and CMR scanning. Co-morbidities, such as arterial hypertension (resting systolic blood pressure > 140 mmHg or diastolic > 90 mmHg or patients already under medical treatment for elevated blood pressure) and diabetes mellitus, were common. Most patients were receiving β-blockers and RAAS inhibitors and had received this treatment for > 12 months prior to CMR.

**Table 2:** Characteristics of the patient population (n = 193).

	All patients	Absence of substantial viability				Substantial Viability	
	**(n = 193)**	**All patients** **(n = 86)**	**LVEF** **< median** **(n = 43)**	**LVEF** **> median** **(n = 43)**	***P1***	**(n = 107)**	***P2***

Age, years	70 (63-75)	69 (59-76)	69 (61-76)	66 (59-76)	0.534	70 (65-75)	0.283
Male sex	167 (87%)	77 (90%)	37 (86%)	40 (93%)	0.291	90 (84%)	0.273
BMI (kg/m^2^)	28 (25-31)	27 (25-31)	29 (25-32)	26 (24-30)	0.111	28 (25-31)	0.773
Systolic blood pressure mmHg)	123 (111-140)	132 (110-148)	117 (102-145)	136 (123-149)	0.002	120 (111-134)	0.084
Diastolic blood pressure (mmHg)	73 (65-80)	74 (64-84)	72 (61-80)	74 (66-86)	0.296	72 (65-80)	0.641
Heart rate (bpm)	65 (59-75)	63 (58-70)	64 (58-74)	62 (57-68)	0.308	66 (59-76)	0.057
**NYHA classification**					**0.022**		**0.357**
NYHA class I	32 (17%)	12 (14%)	3 (14%)	9 (21%)		20 (19%)	
NYHA class II	124 (64%)	60 (70%)	29 (67%)	31 (72%)		64 (60%)	
NYHA class III	37 (19%)	14 (16%)	11 (26%)	3 (7%)		23 (21%)	
Previous infarct	129 (67%)	62 (72%)	34 (79%)	28 (65%)	0.149	67 (63%)	0.165
Previous revascularisation	71 (37%)	29 (34%)	15 (35%)	14 (33%)	0.820	42 (40%)	0.428
**Co-morbidities:**							
History of hypertension	56 (29%)	24 (28%)	10 (23%)	14 (33%)	0.336	32 (30%)	0.761
Diabetes mellitus	36 (19%)	13 (15%)	5 (12%)	8 (19%)	0.366	23 (22%)	0.258
**Medications:**							
β-blockers	167 (87%)	82 (95%)	42 (98%)	40 (93%)	0.306	85 (79%)	0.001
RAAS inhibitors	175 (91%)	83 (97%)	41 (95%)	42 (98%)	0.557	92 (86%)	0.012
Diuretics	130 (67%)	55 (64%)	30 (70%)	25 (58%)	0.261	75 (70%)	0.366
Spironolactone	53 (28%)	21 (24%)	16 (37%)	5 (12%)	0.006	32 (30%)	0.396
Digoxin	18 (9%)	6 (7%)	4 (9%)	2 (5%)	0.397	12 (10%)	0.314
**CMR measurements**							
LVEF	34 (27-43)	41 (29-51)	29 (24-38)	50 (45-57)		31 (24-35)	< 0.0001
LVEDV index	117 (95-150)	103 (84-127)	126 (101-155)	91 (69-104)	< 0.0001	129 (111-156)	< 0.0001
LVESV index	78 (55-110)	57 (41-83)	82 (69-113)	42 (32-54)	< 0.0001	89 (72-117)	< 0.0001
LVMM index	82 (71-101)	80 (68-97)	91 (73-106)	75 (65-84)	0.002	85 (74-101)	0.120
**Segmental analysis**							
Segments	3281	1462	731	731		1819	
Dysfunctional Segments	1759 (53%)	527 (36%)	154 (21%)	373 (51%)	< 0.0001	1232 (68%)	
Dysfunctional segments without LGE	581 (18%)	31 (2%)	14 (2%)	17 (2%)	0.884	550 (30%)	
Dysfunctional segments with LGE	1178 (36%)	496 (34%)	140 (19%)	356 (49%)	< 0.0001	682 (37%)	
Dysfunctional segments with > 50% LGE	559 (17%)	313 (21%)	65 (9%)	248 (34%)	< 0.0001	246 (14%)	
Segments with normal function - no LGE	1326 (40%)	849 (58%)	513 (70%)	336 (46%)	< 0.0001	477 (26%)	
Segments with normal function and LGE	98 (3%)	43 (3%)	32 (4%)	11 (2%)	0.283	55 (3%)	

Data are expressed as median and interquartile range (IQR) or as number and percentage (%) of patients.

*P_1 _*value represents the significance of differences between the subgroups of patients with no substantial viability and LVEF > median (41%) and those with LVEF < median

*P_2 _*value expresses comparison between the groups with significant viability and no significant viability

LV, left ventricular; EF, ejection fraction; BMI, body mass index; NYHA, New York Heart Association; RAAS, rennin-angiotensin-aldosterone system; EDV, end-diastolic volume; ESV, end-systolic volume; MM, myocardial mass; LGE, late gadolinium enhancement.

**Figure 1:**
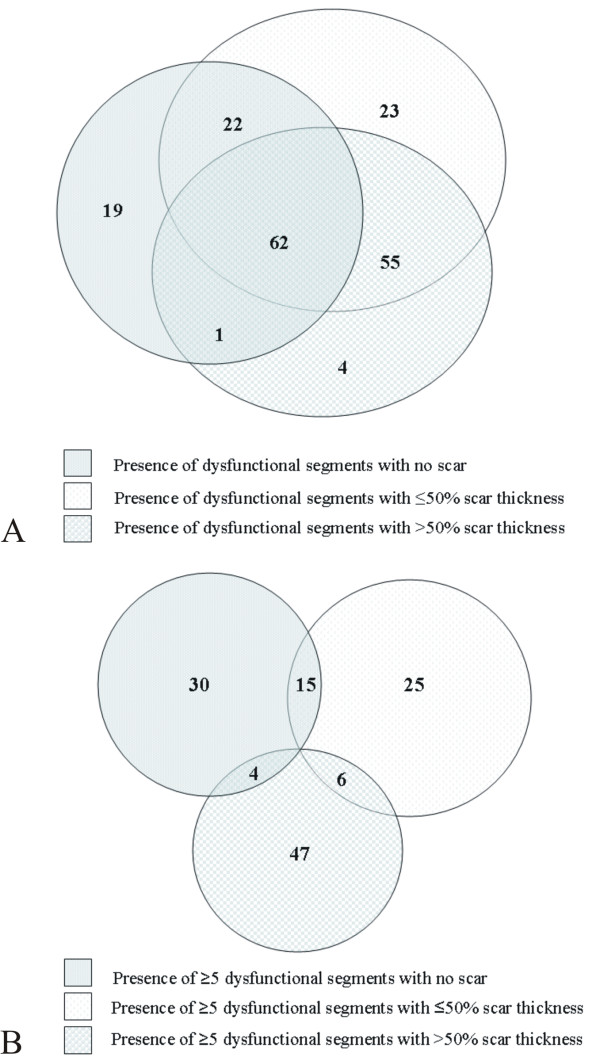
**Two Vein diagrams of which the first shows the presence of dysfunctional segments with > 50%, ≤50% and without late gadolinium enhancement (LGE) (A), and the second the occurrence of ≥5 dysfunctional segments with > 50%, ≤50% and no LGE (B) in the studied population**.

On CMR, 186 patients had at least one dysfunctional segment while seven patients had normal systolic function. LGE was observed in 169 (88%) patients. Substantial myocardial viability, that is ≥5 dysfunctional segments with ≤50% LGE, was found in 107 (55%) patients of whom 49 (25%) had ≥5 dysfunctional segments with no scar and 25 (13%) patients also had ≥5 dysfunctional segments with > 50% LGE. 64 (33%) patients had ≥5 dysfunctional segments with ≤25% scar thickness. Dysfunctional segments without LGE were not uncommon, with 104 patients (53%) having at least one such segment (Figure 1).

**Figure 2:**
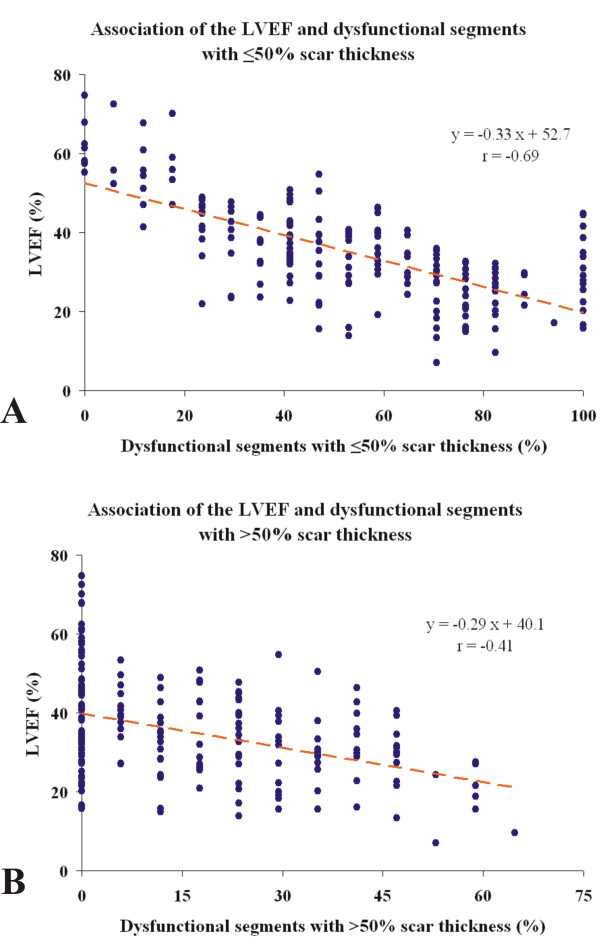
**Associations between left ventricular ejection fraction (LVEF) and dysfunctional segments with ≤50% and > 50% scar thickness**.

Table 2 illustrates differences between the subgroups of patients with and without substantial viability. Patients with substantial viability had to have ≥5 dysfunctional segments, so the proportion of dysfunctional segments was higher in this group than in those without substantial viability (68% *v *36%). Consequently, they had more severe LV systolic dysfunction and dilation and were less likely to tolerate treatment with a RAAS inhibitor and a β-blocker. In other words, patients with more severe LV dysfunction were more likely to have substantial viability (Figure 2). On the other hand the patients without substantial viability appeared to constitute an inhomogeneous group. The subgroup of patients with a LVEF below the median (< 41%) had a considerable proportion of scarred and dysfunctional myocardium while the group with LVEF > 41% had few segments with myocardial dysfunction or scar, consistent with recovery of ventricular function in response to medical therapy.

57 patients (29%) had ≥ 5 segments with scar thickness > 50% but 2 out of 5 of them (24 patients) also had ≥ 5 dysfunctional segments with no or ≤ 50% partial thickness scars. Compared to the other patients they had lower LVEF (29% (22%-34%) *v *37% (29%-45%), *P *< 0.0001) and increased LVEDV index (131 ml/m^2 ^(112 ml/m^2^-173 ml/m^2^) *v *114 ml/m^2 ^(92 ml/m^2^-145 ml/m^2^), *P *= 0.001) and LVESV index (94 ml/m^2 ^(75 ml/m^2^-126 ml/m^2^) *v *72 ml/m^2 ^(51 ml/m^2^-100 ml/m^2^), *P*< 0.0001) but there were no differences in the LVMM index (82 g/m^2 ^(70 g/m^2^-97 g/m^2^) *v *82 g/m^2 ^(74 g/m^2^-101 g/m^2^), *P *= 0.507).

**Figure 3:**
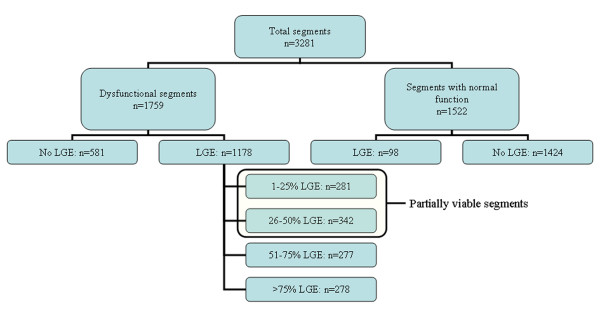
**Prevalence of myocardial segments with and without late gadolinium enhancement (LGE)**.

The prevalence of LGE on a per segment basis is shown in Figure 3. Half of the myocardial segments were dysfunctional. Few segments with normal contractile function (6%) showed LGE (predominantly ≤25% of wall thickness), while two thirds of segments with impaired function demonstrated at least some LGE. Of 1759 segments with contractile dysfunction, 581 had no evidence of myocardial scar, 623 had ≤50%, and 555 segments had > 50% scar thickness.

**Figure 4:**
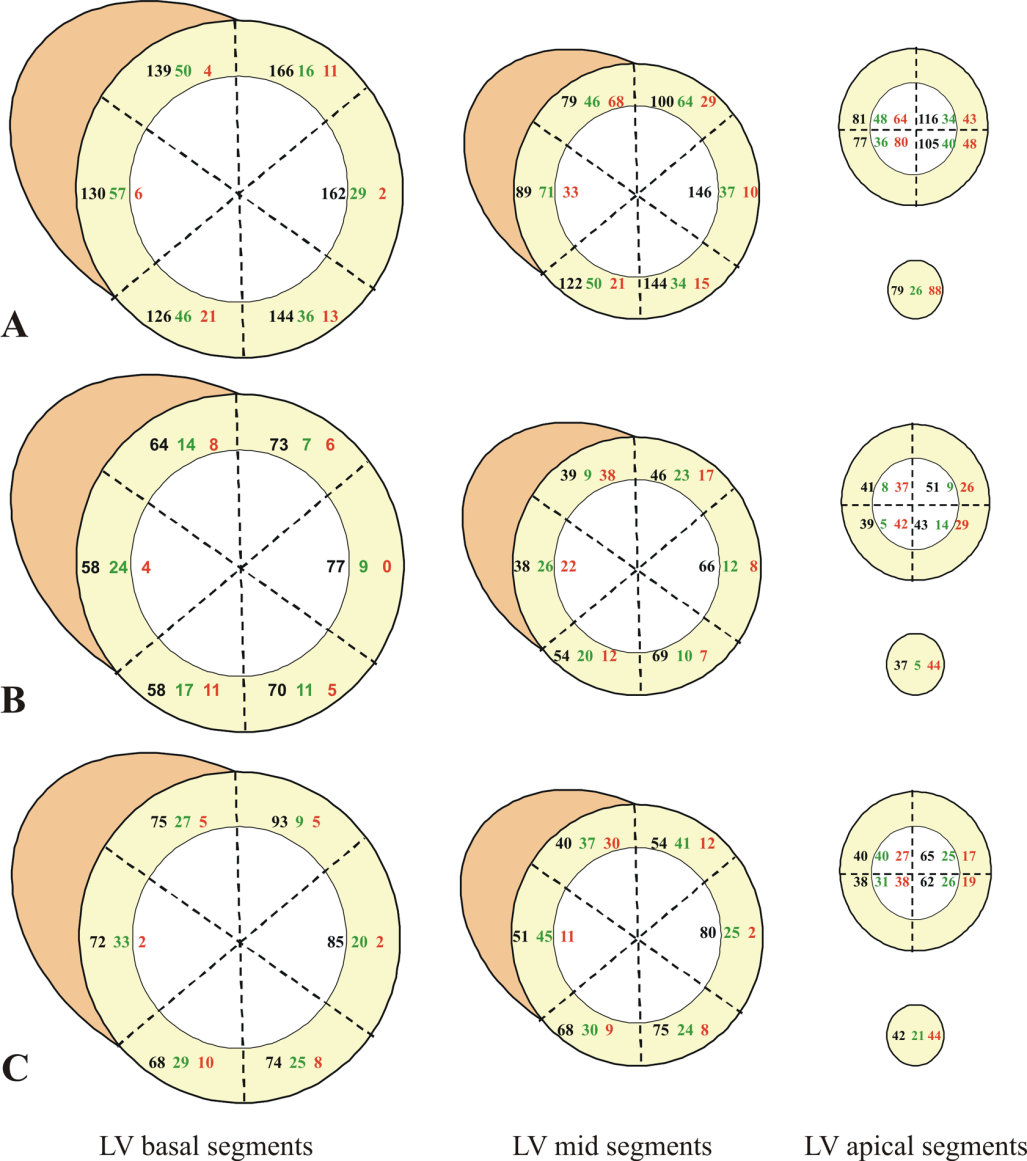
**Scar distribution in the studied population (A), in the group without substantial viability (B) and in the group with substantial viability (C)**. The black numbers correspond to the left ventricular (LV) segments with no late gadolinium enhancement (LGE), the green to the segments with scar thickness ≤50% and the red to the segments with a scar thickness > 50%.

Figure 4 shows the distribution of scar in the studied population. Myocardial scars were more common in apical and septal regions and less common in the LV free wall (occurrence 54% *v *25%, *P *= 0.002). These gradients in distribution were true for partial thickness (≤50% scar thickness) and extensive (> 50%) scars, but more extreme for the latter group (25% *v *7%, *P *= 0.030).

## Discussion

There is still uncertainty as to the proportion of patients with CHF due to IHD who have a substantial volume of viable but dysfunctional myocardium and who might, therefore have recoverable myocardial function. It is difficult to compare studies because they used different imaging methods, different segmental models and different definitions for the number of affected segments required to declare a substantial problem. In previous echocardiographic or radionuclide studies which specified that a substantial (e.g. 4/16 or 5/19 segments) number of such segments had to be present, the proportion of patients with a substantial amount of viable but dysfunctional myocardium ranged from 27% to 61% [7,19-21].

The inability of these techniques to distinguish the relative amounts of scar and viable tissue may limit their use in predicting the recovery of dysfunctional myocardium in response to therapy. Nuclear imaging methods detect viability by assessing perfusion, cell membrane integrity and metabolism but have limited spatial resolution and do not image scar directly [22]. Two small studies have compared the diagnostic accuracy of positron emission tomography and CMR with LGE and one showed that the latter is superior to nuclear imaging in detecting non-viable segments while the other found no difference between the two techniques [23,24]. No studies have compared the diagnostic accuracy of stress echocardiography and CMR with LGE but the CMR is likely to be superior in patients with atrial fibrillation and in those with poor acoustic windows [25]. In addition, there is pathological evidence that hibernating tissue can lose its contractile apparatus and therefore its ability to thicken in response to inotropic stimuli [26]. Thus, echocardiography may not be as reliable method as CMR in assessing myocardial viability. In contrast, CMR supplemented by contrast studies permits the accurate and reproducible assessment of contractility and scar with a single imaging technique [10-12].

Our study highlights the complexity of the myocardial substrate in patients with CHF and IHD. Moderate to severe contractile dysfunction was most commonly associated with partial thickness scar, with slightly more than half of the patients having five or more segments affected. Contractile dysfunction in the absence of underlying scar was also commonly observed, a disturbance of myocardial metabolism most likely induced by ischaemia, hibernation or stunning. Finally, extensive (> 50%) scar was also frequent and affected one third (32%) of the dysfunctional segments. There is likely to be an added complexity in segments with partial thickness scar, where contractile dysfunction will reflect not only the extent of the scar but also the contractility of the remaining myocardium. Ischaemia, hibernation and stunning of the residual myocardium is likely to be common in these segments.

Some meta-analyses have shown that revascularisation in patients with viable myocardium improves outcome [27]. On the other hand two randomised control trials that have been recently published showed a neutral effect of revascularisation on prognosis. Unfortunately, the Heart Failure Revascularisation Trial (HEART) (in which patients with LV systolic dysfunction and a substantial volume of viable myocardium were randomised to medical treatment or revascularisation) was stopped early due to problems with recruitment and funding [28]. Only 138 patients were enrolled. There were no differences in all cause mortality, quality of life or (in a sub-study) systolic function as measured by CMR. However, the study was underpowered and thus the final results needed to be further confirmed [29]. The second trial, STICH (Surgical Treatment for Ischemic Heart Failure), showed no effect of revascularisation on death, but perhaps a modest effect on cardiovascular death or hospitalisations [30]. A sub-study of STICH trial examined the prognostic effect of revascularisation in patients with substantial viability [31]. This included 601 patients (487 with substantial viable myocardium) with a LVEF ≤35% who were followed-up for 5 years. The reported results demonstrated that revascularisation did not appear to improve outcomes (total mortality, cardiovascular mortality or the combined end-point total mortality/hospitalisation due to cardiovascular causes). Though this sub-study provides evidence that revascularisation has a neutral impact on hard end-points it remains unclear whether it should be implemented in patients suffering from CHF with substantial viability to restore myocardial contractility, reduce heart failure symptoms and improve quality of life.

Ultimately, assessing the extent to which LV dysfunction is due to myocardial scar may be more important than trying to assess the potential for functional recovery when assessing a patient for revascularisation. With existing therapies, myocardial scar reflects irreversible damage. Successful revascularisation might not only eliminate myocardial ischaemia and stunning and resuscitate hibernating myocardium but also reduce the risk to viable myocardium, whether or not it is dysfunctional, from further coronary occlusions. The potential of attempted revascularisation to cause irreversible myocardial damage, to which hibernating myocardial may be more prone, should also be considered [32].

Our results may also be important for the selection of patients for CRT. Extensive myocardial scar, perhaps especially when it affects the postero-lateral wall of the LV predicts a lower chance of recovery of ventricular function with CRT [13,14]. This may reduce the functional response to CRT but it is not clear whether it also translates into a lesser impact in terms of prognosis [33]. However, it is reassuring that transmural scar in the postero-lateral wall was relatively uncommon in this population as only 8% of the patients had scar > 50% and only 4% of the patients had > 75% scar in this region.

### Limitations

There was a delay between the initial echocardiographic assessment of cardiac function and CMR. During that period, treatment with RAAS inhibitors and β-blockers were initiated or increased, which may have led to recovery of function in viable myocardial segments. Accordingly, this report will underestimate the prevalence of viable but dysfunctional myocardial segments in a treatment naïve population, although it is still a good estimate of the proportion of segments with extensive scar.

Although the evaluation of the contractile function of the myocardial segments was based on wall thickness it is possible that tethering or reciprocal changes caused by neighboring dyssynchronous segments could lead to inaccurate estimations. By convention, a myocardial segment was considered viable if ≤50% of the segment showed LGE. However, a strict definition of "viability" requires that the segment's contractility improves after revascularisation. As our patients are not routinely revascularised in the absence of angina, we cannot be certain that the segments we have labeled "viable" meet this definition. Although available, the end-diastolic wall thickness of the studied segments was not included in our analysis as the conventional cut-off value of < 5.5 mm is not accurate in excluding functional recovery after revascularisation [34,35]. A significant limitation of this study is the absence of CMR with low-dose dobutamine stress testing that would allow more reliable identification of segments that would improve their function after revascularisation (especially in cases with intermediate extent of scar tissue) [36].

We used the cut-off of 5 segments to define "substantial" myocardial viability since observational studies show that functional improvement following revascularisation is more likely if a third of LV segments show contractile dysfunction with viability [17]. However, recently Pegg et al. demonstrated that myocardial recovery after revascularisation depends not only on the number of the segments with partial scar thickness but also on the number of the normal segments [6]. Considering that this was a small study (only 33 patients were included) it has been decided to use a cut-off (≥5 segments) value already implemented in larger studies, albeit that these used stress echocardiography to define substantial viability [31,37,38]. We acknowledge the limitation of extrapolating this methodology to the present CMR based study and recognize the need for further research to test and refine these criteria preferably in the context of large randomised trials of relevant interventions.

## Conclusions

Viable but dysfunctional myocardial segments in patients with CHF due to LV systolic dysfunction and IHD remain common despite contemporary medical therapy and frequently reflect two different pathologies, partial thickness scar and contractile dysfunction, that may have different prognostic and therapeutic implications. The extent of myocardial scar, which currently reflects irreversible damage, may be a useful guide to the likely extent of recovery of ventricular function with pharmacological interventions and revascularisation.

## Competing interests

The authors declare that they have no competing interests.

## Authors' contributions

CVB involved in data analysis and drafting the manuscript; NPN designed the study and involved in drafting the manuscript; HPL RdeS and NS contributed in data collection and interpretation, EIL analyzed the CMR images, ACT, MFA and SG revised the manuscript for important intellectual content while KW interpreted the study findings and ALC and JGFC were the general supervisors of this study. All authors have read and approved the manuscript.
